# Progress in Cerebral, Spinal, and Nerve Surgery

**Published:** 1904-03-19

**Authors:** 


*(To be continued.)*

### Progress in Cerebral, Spinal, and Nerve Surgery.

**Fracture of the Atlas and Odontoid Process without Symptoms.**—Scott[1] reports this case from Roorkee, India. The patient stated that he had been struck across the back of the neck with a heavy stick and both his arms had been injured. There was pain and stiffness in the neck, but no bruising. There was a Colles' fracture in each arm, and under the anæsthetic administered for the reduction of these the neck was fairly forcibly rotated, but nothing could be felt. The patient died of tetanus on the tenth day from the injury, there having been no further symptoms referable to the neck. At the autopsy the anterior and posterior arches of the atlas were found fractured, and the odontoid process snapped off at its base and lying loose, but held in place by the transverse ligament.

**Recovery from Fracture of the Fifth Cervical Vertebra.**—Tinley and Jones[2] relate this case. A man fell 40 feet from a bridge on to a slope below. He was carried to his home, when some time after he was found to be semi-conscious with complete loss of power in the legs and some loss in the arms. There was pain in the back of the neck and the pupils were contracted. The following morning he had retention of urine. There was complete paralysis of the lower limbs with loss of superficial and deep reflexes. Priapism was present. The intercostals were paralysed and respiration difficult. The right arm was abducted; the elbow flexed and hand supinated. The fingers and wrist were motionless on the right side. This was less marked on the left side, but in a day or two became as complete as on the right side. Four days after the accident he had incontinence of urine and bedsores began to develop. The most tender spot was over the fifth cervical vertebra. His bedsores having got much worse and his general condition very critical, he was removed to a cottage hospital, where for some months his condition remained much the same. The superficial reflexes returned, however, and he became hypersensitive, the patellar reflexes were exaggerated and he developed ankle clonus. About seven months after the accident he began voluntarily to move his feet and later to raise his knees, and ten months after began to move his arms a little. Faradism and massage were applied, and about a year from the accident he could take a few steps with help, and a month later could walk a little, but with a distinctly spastic gait. His condition now was as follows: He had good movement at the ankles, knees, and hips, and greatly increased knee-jerks; superficial reflexes normal; intercostal muscles still lacked power. He had control over his urine, but occasional want of control over his motions. Some of his fingers could be flexed and extended, others were ankylosed. Movements of left shoulder and elbow were nearly normal; the right elbow was nearly fixed at a right angle. Over the fifth cervical vertebra was a boss of bone as big as a walnut. He carried his head bent forward and stiff.

**Some Points relating to Rigidity of the Spine.**—Bennett[3] in a clinical lecture points out that rigidity of the spine is not always evidence of disease, and that there are two kinds of rigidity, the absolute and the hesitating or stammering kind. To examine an infant for rigidity, the child should be placed on its back on a couch and the hand placed beneath the spine and an attempt made to raise the patient, the ordinary method of examining an adult by bending down being inapplicable. The fact that rigidity disappears under an anæsthetic is no certain sign that disease is absent. Rigidity may involve the whole length of the column or only a limited portion. A case is related of a boy of 14 stiff from the sacrum to the cervical region, and supposed to have spinal caries. Careful examination detected some "stammering" in the muscles, and the patient being kept in the bent position *for some minutes* the muscles began to give way. The rigidity was all muscular, and was due to neuro-mimesis. He had spent some time with a relative under treatment for caries with a jacket. Massage and exercises soon cured him. In the presence of absolute rigidity, independent of muscular contraction, it should not be taken for granted that there is organic disease. A case is mentioned of a girl of 14 with absolute rigidity of the lumbar and lower two dorsal vertebræ, and supposed to be the subject of spinal caries. There were no other symptoms, and never had been, and she was in perfect health and able to indulge in all outdoor games. An examination of the father and a younger sister showed rigidity in the same parts. It was a case of congenital spinal rigidity. This congenital peculiarity may affect other parts of the spine, and should be borne in mind in estimating the importance of painless spinal rigidity. As the presence of spinal rigidity is no positive evidence of organic disease, so, on the other hand, the absence of rigidity is no positive evidence of the absence of disease. A case is quoted of a tubercular focus in the front of one of the vertebral bodies with a large psoas abscess and no rigidity. The causes of spinal rigidity are numerous, and vary at different periods of life. They may be thus classified: 1. *In infancy and early childhood* the principal causes are injury, adenitis and glandular abscess, worms and tubercular disease. 2. *From puberty to the age of twenty-five*, injury, adenitis and glandular abscess, early osteo-arthritis, neuro-mimesis, the congenital peculiarity mentioned above, advanced scoliosis, myositis ossificans and tubercle—the most important. 3. *From the age of twenty five to fifty five*, adenitis and glandular abscess, tubercle, malignant disease, hydatid cysts, gumma, aneurisms, osteo-arthritis, congenital peculiarity, injury and myositis ossificans. 4. *From fifty-five onwards*, osteo-arthritis, spontaneous ankylosis, the ordinary rigidity of old age, secondary carcinoma, injury and senile tubercle.

[1] Brit. Med. Jour., Jan. 30, 1904. [2] Lancet, Nov. 28, 1903. [3] Clin. Jour., Nov. 11, 1903.

